# Immunogenicity Rates after SARS-CoV-2 Three-Dose Vaccination in Patients under Dialysis: A Systematic Review and Meta-Analysis

**DOI:** 10.3390/vaccines10122070

**Published:** 2022-12-02

**Authors:** Xiuhong Yang, Hua Zhang, Wenjing Bao, Shunkun Fu, Huimin Jin

**Affiliations:** Division of Nephrology, Shanghai Pudong Hospital, Fudan University, Pudong Medical Center, 2800 Gong Wei Road, Shanghai 201399, China

**Keywords:** meta-analysis, 3-dose of SARS-CoV-2 vaccine, hemodialysis, peritoneal dialysis, immunogenicity rates

## Abstract

Background: Considering the indeterminate effects following the administration of three doses of the SARS-CoV-2 vaccine to patients under dialysis, the present study aimed to evaluate the immunogenicity rates of patients who received the three-dose vaccine. Methods: MEDLINE, Web of Science, EMBASE, ClinicalTrials.gov, and the Cochrane Central Register for Controlled Trials were searched to select the relevant literature to perform the present review. We included randomized controlled trials, non-randomized trials, prospective, observational cohort, and case-control studies to assess the humoral and cellular immune responses following the administration of the three-dose SARS-CoV-2 vaccine to patients receiving dialysis. Results: Overall, 38 studies are included in the meta-analysis presented in this paper. For patients on dialysis, the overall humoral antibody response rate is 97% following three doses of mRNA or viral vector vaccines and 100% following four doses of the SARS-CoV-2 vaccine. A subgroup analysis shows that the antibody response rate is 96% for patients on hemodialysis (HD) and 100% for those receiving peritoneal dialysis (PD). The antibody response rate in the different immunogen-vaccinated groups tends to be higher than that in the same immunogen-vaccinated group (99% vs. 96%). For those who exhibit no response following two doses of the vaccine, the third and fourth doses can elevate the antibody response rate to 81%, and that number for low responders increases to 96%. However, the pooled results obtained from the relatively few trials conducted indicate that the positive T-cell response rate only increases to 59% following three doses of the vaccine. The antibody response rate is not different between dialysis and non-dialysis groups (relative risk = 0.95, 95% CI 0.90–1.02) following three doses of the vaccine. The relative risks for a SARS-CoV-2 breakthrough infection, all-cause mortality, and hospital admissions are 0.59 (95% CI 0.30–1.04), 0.63 (95% CI 0.35–1.12), and 0.53 (95% CI 0.37–0.74), respectively, when comparing three doses with two doses of the vaccine administered to the dialysis population. Conclusions: The third or fourth dose of the SARS-CoV-2 vaccine significantly increases the immunogenicity rates in dialysis patients, and this beneficial effect does not vary with the type of vaccine (the same or different immunogen vaccination), dialysis modality (HD or PD), or previous low response following the administration two doses of the vaccine. We believe that healthcare workers should encourage patients receiving dialysis to receive a third or fourth vaccine dose to strengthen their immunity against SARS-CoV-2.

## 1. Introduction

Severe acute respiratory syndrome coronavirus 2 (SARS-CoV-2) has been a pandemic for more than two years and led to millions of confirmed disease cases and numerous deaths. Clinical trials show that the Pfizer and Moderna vaccines are 95.0% effective, and the Johnson & Johnson vaccine is 66.0% effective in protecting the general population against SARS-CoV-2 infection [1]. Patients on hemodialysis (HD) or peritoneal dialysis (PD) have impaired and inferior immune function, and are at a high risk of mortality if infected with SARS-CoV-2 [2,3]. The efficacy and safety of the SARS-CoV-2 vaccine in patients receiving dialysis has been highly debated in the field of study. It has been observed that there is a high response rate to the SARS-CoV-2 vaccine in the dialysis population after receiving two doses of the SARS-CoV-2 vaccine [4,5]. A previous meta-analysis conducted in 32 studies demonstrated that the overall immunogenicity rate to the SARS-CoV-2 vaccine in the dialysis population was 86% (95% CI, 81–89%), and had a low response rate after the first (RR = 0.61; 95% CI 0.47–0.79) and second (RR = 0.88; 95% CI 0.82–0.93) doses of the vaccine when compared to the non-dialysis controls [6]. As the virus evolved, a third-dose shot attracted further attention. In the non-dialysis population, it was observed that the third-dose vaccines demonstrated an overall increased reactogenicity result: mRNA-1273 after ChAd/ChAd or BNT/BNT, and ChAd and Ad26 after BNT/BNT after receiving a third dose (booster) of the vaccine [7]. There are reports that claim that the administration of a third-dose vaccine within 1 to 2 months of receiving a prime-boost vaccination could increase antibody levels, particularly in patients with poor initial responses (such as patients receiving dialysis) [8]. However, due to the small sample sizes used in previous studies, there is not enough evidence to support the recommendation of a third SARS-CoV-2 vaccine dose to complete an ideal seroconversion after a two-dose prime regimen in patients on dialysis. Therefore, the meta-analysis presented in this study is conducted to confirm further the hypothesis that a third or fourth dose of the vaccine could promote seroconversion and increase the presence of neutralizing antibodies in dialysis patients.

## 2. Methods

### 2.1. Search Strategy and Study Selection

We searched the relevant studies using the following databases: Web of Science, MEDLINE (from 1 January 2020 to 31 August 2022), and Embase (from 1 January 2020 to 31 August 2022), using the following keywords: dialysis AND (3-dose vaccine OR 4-dose vaccine OR boost vaccine) AND (SARS-CoV-2 vaccine, ChAdOx1 nCoV-19, Oxford–AstraZeneca, mRNA vaccines, BNT162b2, OR mRNA-1273). The language was restricted to English in our meta-analysis. The literature collection procedure is presented in Figure S1.

### 2.2. Inclusion and Exclusion Criteria

We studied the following areas: (1) patients receiving HD or PD; (2) randomized controlled trials, non-randomized trials, or prospective, retrospective observational studies; and (3) reports of post-vaccination antibody response rates. The following studies were excluded: (1) different publications analyzing the same population or duplicates, and (2) no reported post-vaccination antibody response rates.

### 2.3. Data Collection

The data (risk ratios, 95% confidence intervals, and response rates) were collected preferentially and independently extracted by the three authors. We also extracted all the study characteristics (i.e., study design, vaccine type, and kidney-replacement modality). Any disagreements were resolved through a discussion or consultation with the other authors.

### 2.4. Heterogeneity Assessment

Heterogeneity was assessed using Cochran’s Q test and I^2^ statistics. The study was judged as heterogeneous if the *p*-value was <0.1 (Cochran’s Q). If one study presented I^2^ values < 50%, we considered it as non-heterogeneous, and a fixed-effects model was used in the analysis; studies with I^2^ > 50% were considered heterogeneous and were analyzed using a random-effects model.

### 2.5. Quality Assessment

Two researchers analyzed the quality of each study using the Newcastle–Ottawa scale, presenting a maximum of nine stars to a study based on patient selection, study design, comparability, exposure, and outcome. A study was considered to be of “high quality” if it scored nine stars on this scale and “medium quality” if it scored seven or eight stars. Discrepancies were resolved through discussions with the other researchers.

### 2.6. Outcome Measures

The primary outcomes were defined as post-vaccination antibody response rates (humoral immunity) and positive T-cell response (cellular immunity) among patients on dialysis. The secondary outcomes were defined as incidences of a breakthrough SARS-CoV-2 infection, admission, and all-cause death in dialysis patients who received a three-dose vaccine compared with those who received two doses or less of the vaccine.

### 2.7. Statistical Analyses

We used STATA version 14.0 (StataCorp, College Station, TX, USA) to analyze the data. The primary outcomes were pooled using the DerSimonian–Laird random-effects model. Heterogeneity was assessed by using the I^2^ statistic. To explore the potential source of heterogeneity, we conducted several subgroup analyses using mixed-effects models, including (1) vaccine type; (2) vaccine dose; (3) kidney-replacement-therapy modality, including HD or PD; and (4) same vaccine type as in the primary vaccination or not.

The risk ratios (RRs) for a breakthrough infection, admission, and all-cause death of the secondary outcomes were extracted from the studies. We examined the presence of publication bias by using Egger’s test. Additionally, we performed a sensitivity analysis to evaluate the influence of the outcomes. *p* < 0.05 was statistically significant.

## 3. Results

### 3.1. Study Selection and Characteristics

Overall, 38 studies were included [9,10,11,12,13,14,15,16,17,18,19,20,21,22,23,24,25,26,27,28,29,30,31,32,33,34,35,36,37,38,39,40,41,42,43,44,45,46] in the meta-analysis (Figure S1). Table 1 presents the characteristics of the 38 included studies.

### 3.2. Post-Vaccination Humoral Immunogenicity Rates

**Figure 1:**
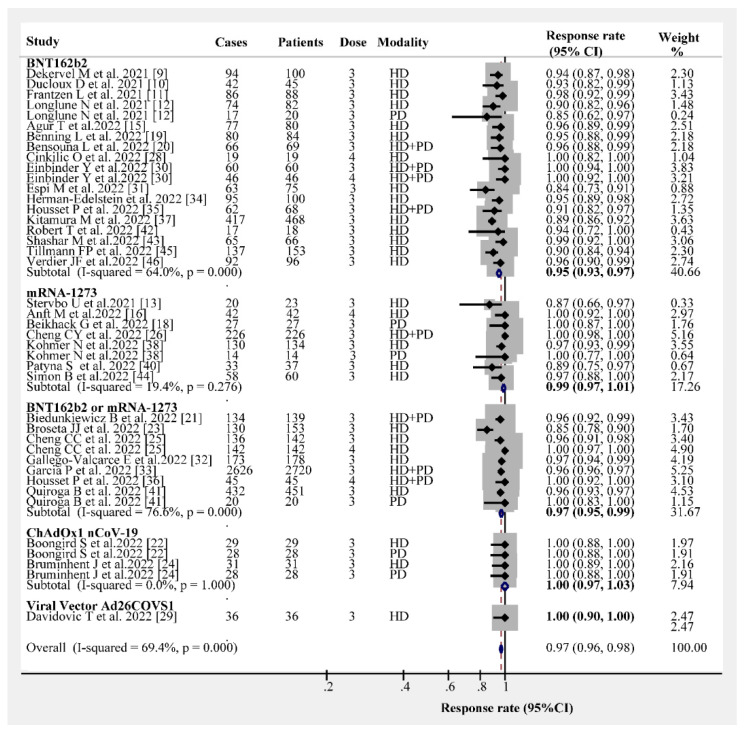
Forest plots of humoral immunogenicity rates vs. vaccine type [9,10,11,12,13,15,16,18,19,20,21,22,23,24,25,26,28,29,30,31,32,33,34,35,36,37,38,40,41,42,43,44,45,46].

**Figure 2:**
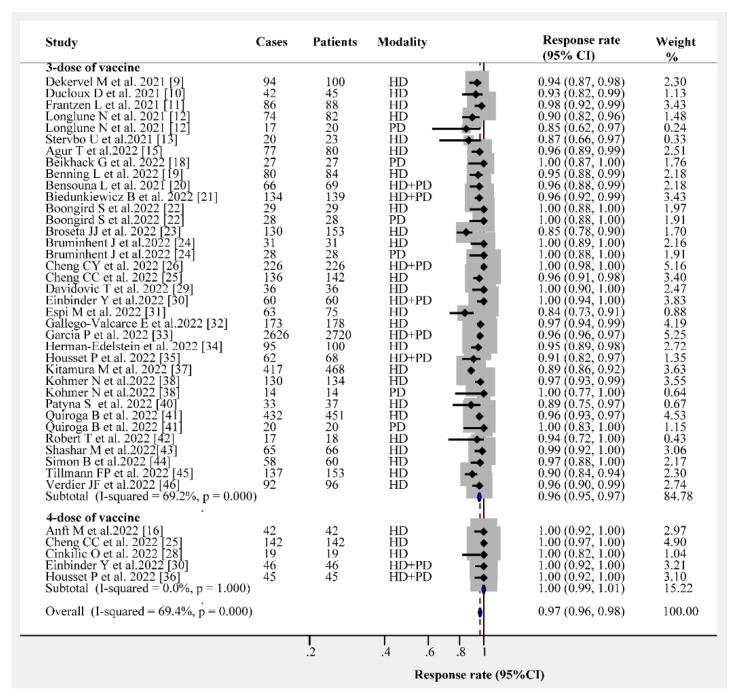
Forest plots of humoral immunogenicity rates vs. the number of vaccine doses [9,10,11,12,13,15,16,18,20,21,22,23,24,25,26,28,29,30,31,32,33,34,35,36,37,38,40,41,42,43,44,45,46].

**Figure 3:**
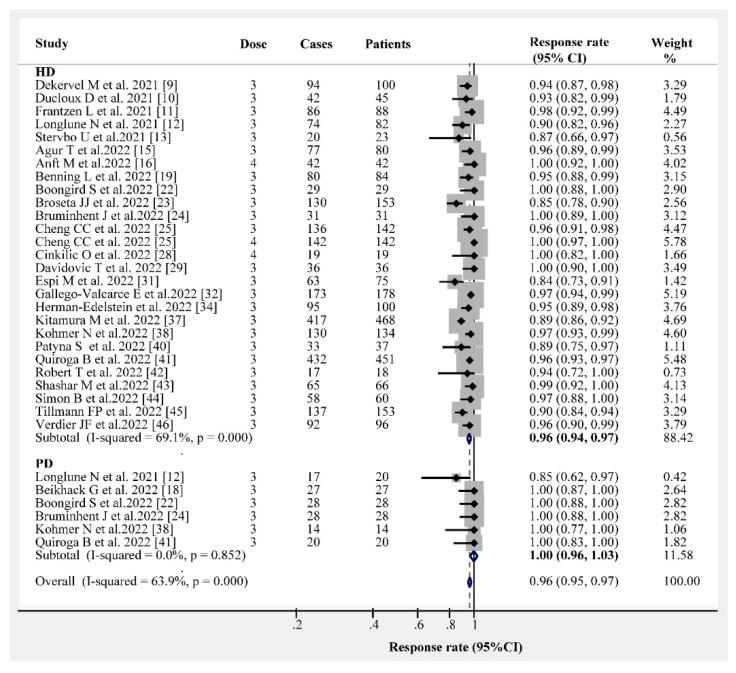
Forest plots of humoral immunogenicity rates vs. different dialysis models (HD or PD) [9,10,11,12,13,15,16,18,19,22,23,24,25,28,29,31,32,34,37,38,40,41,42,43,44,45,46].

**Figure 4:**
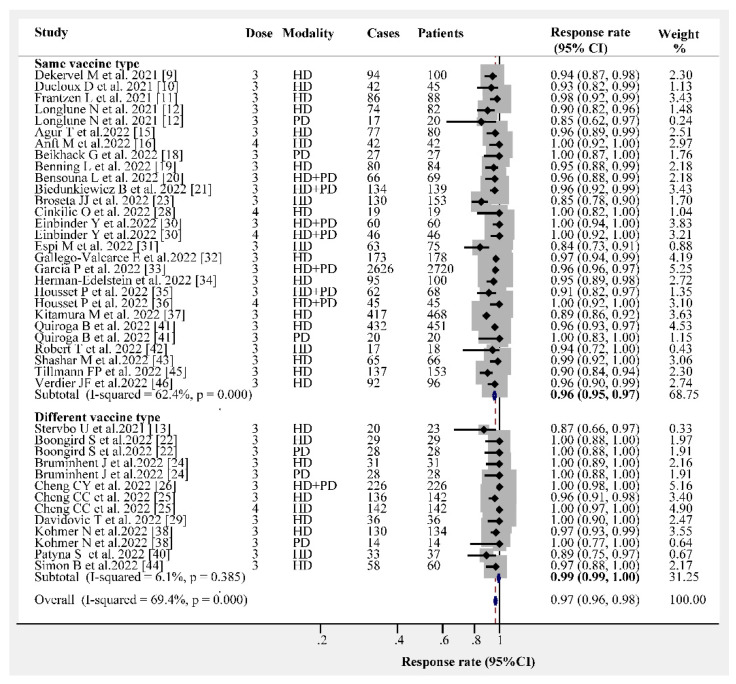
Forest plots of humoral immunogenicity rates vs. homologous/heterologous vaccines [9,10,11,12,13,15,16,18,20,21,22,23,24,25,26,28,29,30,31,32,33,34,35,36,37,38,40,41,42,43,44,45,46].

**Figure 5:**
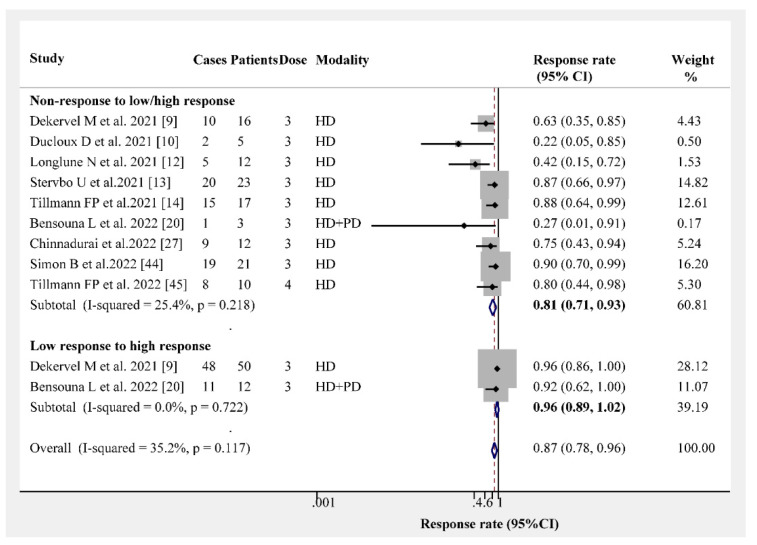
Forest plots of humoral immunogenicity rates vs. non-/low responders [9,10,12,13,14,20,27,44,45].

The overall humoral immunogenicity rate in patients receiving dialysis was 97% (95% CI, 96–98%). We observed that the vaccine type had no effect on the humoral immunogenicity rate, and the response rate following the administration of three- or four-dose vaccines was consistently higher than 95% (Figure 1). The response rates following three- and four-dose vaccine administrations were 96% (95% CI, 95–97%) and 100% (95% CI, 99–100%), respectively (Figure 2). Different dialysis models (HD or PD) may affect the immunogenicity rate; therefore, we separated HD from PD. The response rate of HD patients after receiving the three- and four-dose vaccines was 96% (95% CI, 94–97%) and 100% (95% CI, 96–100%), respectively (Figure 3). The same and different immunogen vaccination regimens potentially evoke different responses in dialysis patients; therefore, we attempted to subgroup those results. The antibody response rate in the same immunogen vaccination regimen group was 96% (95% CI, 95–97%), and the antibody response rate in the different immunogen vaccination regimen group was 99% (95% CI, 99–100%) (Figure 4). For those who were low or had no responders after receiving two doses of the SARS-CoV-2 vaccination, we estimated the antibody response rate after three or four doses. As presented in Figure 5, after three doses of the vaccine, the immunogenicity rates of those who were non-responders increased to 81% (95% CI 71–93%), and the response rate increased to 96% (95% CI 89–100%) in those who were low responders after two doses. We believed that a difference in the immunogenicity rates may exist between patients on dialysis and those that are not on dialysis; therefore, we compared the immunogenicity rates of these two groups further. However, there were no obvious differences in the immunogenicity rates between the two groups after receiving three doses of the vaccine (RR = 0.95, 95% CI 0.90–1.02; Figure S2).

### 3.3. Cellular Immunogenicity Rates

**Figure 6:**
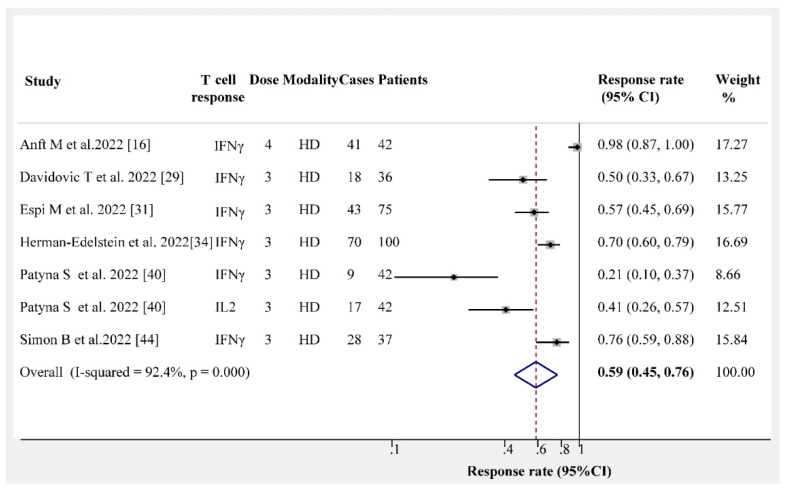
Forest plots of cellular immunogenicity rates [16,29,31,34,40,44].

There were six trials recording the cellular immunogenicity rates. The cellular immune response was measured by the release of interferon-γ or interleukin-2. The pooled results from the six trials indicate that the cellular immunogenicity rate for patients on dialysis is 59% (95% CI, 45–76%) and exhibits high heterogeneity (I^2^ = 92.4%) (Figure 6). This result is significantly lower than that observed in the humoral immunity group (97%) after receiving three or four doses of the vaccine.

### 3.4. RRs for a Breakthrough SARS-CoV-2 Infection, All-Cause Death, and Admission

Only seven trials reported the risks of a SARS-CoV-2 breakthrough infection, all-cause death, and admission in patients on dialysis after receiving three doses of the vaccine. As shown in Figure S3, the rates of a SARS-CoV-2 breakthrough infection and all-cause death are not significantly different between dialysis patients after receiving three or two doses of the vaccine (RR = 0.59, 95% CI 0.3–1.14; RR = 0.63, 95% CI 0.35–1.12; respectively). However, compared to the two-dose-vaccine group, dialysis patients exhibited a decreased risk of hospital admissions after receiving the three-dose vaccines (RR = 0.53, 95% CI 0.37–0.74).

### 3.5. Adverse Events of the SARS-CoV-2 Vaccine

Adverse events, such as fatigue, muscle pain, fever, and injection site pain, were commonly experienced after the administration of the SARS-CoV-2 vaccine. As presented in Figure S4, the incidence rate of fatigue is 37% (95% CI, 21–65%), muscle pain 30% (95% CI, 14–65%), fever 23% (95% CI, 11–46%), injection site pain 49% (95% CI, 28–86%), and others 14% (95% CI, 7–28%).

## 4. Discussion

For the first time, we demonstrated that the third or fourth dose of the same or different SARS-CoV-2 vaccination regimen could evoke seroconversion and elevate neutralizing antibodies, as well as increase the immunogenicity rates for patients receiving dialysis regardless of their response or low-response status after a previously experiencing a two-dose prime-regimen strategy. The data obtained from a meta-analysis of 38 trials indicate that it is necessary to commence a third-dose regimen for patients who exhibit no or low responses to a previously administered two-dose vaccine.

At present, there are several vaccine types that have been approved by the World Health Organization: (1) messenger RNA: BNT 162b2 (Pfizer, Inc., Philadelphia, PA, USA) and mRNA-1273 Moderna vaccines (ModernaTX, Inc., Cambridge, MA, USA); (2) viral vector (adenovirus): ChA-dOx1 (ChAd, AZD 1222, AstraZeneca/Oxford, UK) and Janssen AD 26. COV2. S (Janssen Biotech, Inc., A Janssen Pharmaceutical company, Johnson & Johnson, New Brunswick, NJ, USA); (3) inactivated virus: China National Pharmaceutical Group, Beijing, China and Sinovac Biotech Ltd., Beijing, China; and (4) Novavax (recombinant protein) [47]. It is unclear whether giving the same immunogen for priming and boosting or different immunogens for priming and boosting in SARS-CoV-2 vaccination affects seroconversion. If so, that would influence which immunogen is selected for the third-dose administration. In BBIBP-CorV-vaccinated healthcare personnel, it was observed that both BNT162b2 and BBIBP-CorV vaccines were associated with protection against laboratory-confirmed SARS-CoV-2 infection after the administration of the third dose of the vaccine [48]. Similarly, it was observed that the administration of a third BNT162b2 dose after two BBIBP-CorV doses significantly enhanced both humoral and T-cell-mediated immune responses, which were comparable to those of three BNT162b2 vaccines [49]. With regard to patients on dialysis, few studies have investigated the effect of different vaccines used as the first and second doses. It was observed that the different vaccinations with ChAd/BNT obviously induced stronger and more frequent humoral immunity responses compared to the same vaccinations with BNT/BNT or ChAd/ChAd in SARS-CoV-2-naïve hemodialysis patients [50]. Another study showed that two BNT162/b2 doses followed by one mRNA 1273 dose within 6 months in patients on maintenance dialysis induced elevated titers of SARS-CoV-2 spike antibodies in almost all patients [38].

The mechanism underlying enhanced immunogenicity rates following a third dose of the SARS-CoV-2 vaccine has not been fully elucidated in the literature. Memory B cells may play an important role in modulating the effects of booster vaccine doses. Under physiological conditions, memory B cells produce low levels of antibodies. When antigens invade during a breakthrough infection, these cells undergo clonal expansion and produce antibody-secreting plasma cells as well as germinal central B cells [51]. In a longitudinal cohort of 42 two-dose-vaccinated volunteers, it was observed that a third dose of the mRNA vaccine increased memory B-cell potency and breadth [52]. The study observed that individuals receiving the third dose of an mRNA vaccine developed a diverse memory B-cell repertoire that could rapidly respond to invading antigens and produce abundant antibodies capable of clearing diverse variants, including Omicron. Such observations may partially explain why receiving a third dose of the SARS-CoV-2 vaccine can induce stronger immunogenicity rates.

It is generally accepted that the antibody titer or antibody level against SARS-CoV-2 is consistent with immunization efficiency or protection against breakthrough infections. A study conducted on Japanese and Indian individuals observed that people who had a moderate response level to the SARS-CoV-2 vaccine also had significantly lower rates of SARS-CoV-2 infections compared to those who had not received the vaccine [53,54]. A Spanish nationwide cohort study indicated that the estimated effectiveness of the vaccine was 43.6% when the booster vaccine was administered between 151 and 180 days after complete vaccination, and 52.2% if administered more than 180 days after the primary scheduled completion [55]. Very few studies have investigated the incidence of a breakthrough infection after a third SARS-CoV-2 vaccine dose is administered to patients on dialysis. From several small trials, our pooled results indicate that the number of hospital admissions due to SARS-CoV-2 breakthrough infections after a third vaccine dose is significantly lower in dialysis patients than in those with a partial vaccination; however, there are no differences in SARS-CoV-2 breakthrough infections and all-cause mortality rates. Considering the limited number of reports on this topic, we can infer the preventive effect of a third dose on breakthrough infections after receiving the previous two doses. A large, retrospective, observational study conducted on 142,826 participants on dialysis indicated that the vaccinated patients’ risk of being diagnosed with SARS-CoV-2 infection post-vaccination was significantly lower when compared to the unvaccinated controls, and vaccinated patients were significantly less likely to be hospitalized compared to unvaccinated patients (for BNT162b2, 28.0% versus 43.4%; for mRNA-1273, 37.2% versus 45.6%). Furthermore, vaccinated patients were significantly less likely to die (for BNT162b2, 4.0% versus 12.1%; for mRNA-1273, 5.6% versus 14.5%). Correspondingly, antibodies were detected in 98.1% and 96.0% of patients who received BNT162b2 and mRNA-1273 vaccines, respectively [56]. Another study also observed that a two-dose-completion vaccination was associated with a 75% lower risk of hospital admission and 88% fewer mortality cases compared to unvaccinated dialysis patients [57]. Antibody response is important for predicting breakthrough infections. The pre-breakthrough index of antibodies to the receptor-binding domain (RBD) values was less than 10, while values between 10 and 23 were associated with higher odds for SARS-CoV-2 breakthrough infections in patients receiving dialysis [57]. In this meta-analysis, the pooled immunogenicity rates approached 100% after the third dose of the vaccine was administered to both favorable and poor responders, which indicated that receiving a third dose provides better protection against SARS-CoV-2 infection.

The cellular immune response is also crucial for protecting against infection. The T-cell assays indicated that there were no significant differences between low- and high-antibody groups in healthcare workers following two doses of the BNT162/b2 vaccine [58]. In immunosuppressed patients and those with hematological system diseases, low cellular response rates were also observed following two doses and a booster dose of the SARS-CoV-2 vaccine [59,60]. Whether humoral or cellular immunity plays a more significant role in preventing SARS-CoV-2 infection remains controversial. The incidence of the Omicron infection in 2865 healthy workers two months after receiving the third dose for SARS-CoV-2 indicated that a low IgG peak was associated with Omicron infection, especially among participants aged 65 years and older, and the weakening of IgG and neutralizing antibodies were associated with increased Omicron infection rates regardless of their cellular immunity status [61]. From our pooled trials, cellular immunogenicity rates were observed to be lower than the humoral immunogenicity rates following three doses of the SARS-CoV-2 vaccine in dialysis patients. This phenomenon is similar to that previously mentioned for immunosuppressed individuals, and potentially reflects injured cellular immune responses in dialytic patients. The precise mechanism of impaired cellular immunity remains to be explored in future studies.

Our meta-analysis was subject to several potential limitations. Firstly, there were few trials that reported a breakthrough SARS-CoV-2 infection and all-cause mortality rates after the third dose of the vaccine was administered to patients on dialysis, and most studies only observed the immunogenicity rates following three doses. Although immunogenicity rates and antibody titer or antibody levels are proposed to indirectly reflect the subsequent outcomes, such as breakthrough infection incidence, hospital admissions, disease severity, and mortality, more trials are required to confirm these clinical outcomes in the future. Secondly, relatively few trials indicated that cellular immunity is lower than antibody response in humoral immunity after receiving the third dose of the vaccine for patients on dialysis; however, this conclusion must be confirmed in more trials in the future. Thirdly, different SARS-CoV-2 variants have different breakthrough potentials. For example, a breakthrough infection of the Delta variant could occur at a higher antibody threshold than that of the Alpha variant. However, we could not take different variants into account because of incomplete data on transmitted variants in the literature.

## 5. Conclusions

In conclusion, our meta-analysis revealed that the humoral immunogenicity rates following three doses of the SARS-CoV-2 vaccine being administered to patients receiving dialysis was close to 100%, which is independent of the fact that individuals may be responders, non-responders, or low-responders following two previously administered doses of the vaccine. The pooled results indicate that the cellular immune response is low following a third dose of the vaccine. Subsequent research should investigate whether a third SARS-CoV-2 vaccine dose reduces breakthrough SARS-CoV-2 infection incidence rates, hospital admissions, disease severity, and all-cause mortality rates in patients receiving dialysis.

## Figures and Tables

**Table 1:** Characteristics of the 38 studies.

Study	N	Age (Years)	Sex (Male%)	KPT Modality (N)	Vaccine Type	Homologous or Heterologous Vaccinations	Dose	Prior SARS-CoV-2 Infection	Antibody Outcome	Follow Up
Dekervel M et al., 2021 [9]	100	55–85	57.0	HD	BNT162b2	Homologous	3	No	Anti-S IgG	4 weeks
Ducloux D et al., 2021 [10]	45	69 ± 10	59.0	HD	BNT162b2	Homologous	3	No	Anti-S IgG	4 weeks
Frantzen L et al., 2021 [11]	88	76 (65–83)	73.0	HD	BNT162b2	Homologous	3	No	Anti-S IgG	4 weeks
Longlune N et al., 2021 [12]	102	64 ± 14	75.5	HD 82; PD 20	BNT162b2	Homologous	3	No	Anti-S IgG	4 weeks
Stervbo U et al., 2021 [13]	23	64 (52–73)	68.0	HD	mRNA-1273	Heterologous	3	No	Anti-RBD IgG	4 weeks
Tillmann FP et al., 2021 [14]	17	64 ± 8	64.0	HD	mRNA-1273	Heterologous	3	No	Anti-RBD IgG	4–5 weeks
Agur T et al., 2022 [15]	80	65.2 ± 12.8	63.4	HD	BNT162b2	Homologous	3	No	Anti-RBD IgG	3 weeks
Anft M et al., 2022 [16]	42	81(66–84)	57.1	HD	mRNA-1273	Homologous	4	No	Neutralizing Antibodies; IFNγ	8–9 weeks
Ashby D et al., 2022 [17]	1126	61 (51–80)	59.0	HD	BNT162b2 or mRNA-1273	Homologous or heterologous	3	Mixed	NR	NR
Beikhack G et al., 2022 [18]	27	54.3 (33–76)	63.0	PD	mRNA-1273	Homologous	3	No	Anti-RBD IgG	4 weeks
Benning L et al., 2022 [19]	84	72 (62–79)	69.0	HD	BNT162b2	Homologous	3	No	Anti-S IgG	4 weeks
Bensouna L et al., 2022 [20]	69	68 (53–76)	65.0	HD 38; PD 31	BNT162b2	Homologous	3	No	Anti-S IgG	3 weeks
Biedunkiewicz B et al., 2022 [21]	139	69 (57–75)	63.3	HD 129; PD 10	BNT162b2 or mRNA-1273	Homologous	3	No	Anti-S IgG	2 weeks
Boongird S et al., 2022 [22]	57	18–59	68.4	HD 29; PD 28	ChAdOx1 nCoV-19	Heterologous	3	No	Anti-RBD IgG	2 weeks
Broseta JJ et al., 2022 [23]	153	72.12 ± 14.44	54.2	HD	BNT162b2 or mRNA-1273	Homologous	3	No	Anti-RBD IgG	2 weeks
Bruminhent J et al., 2022 [24]	59	51 (42–54)	58.0	HD 31; PD 28	ChAdOx1 nCoV-19	Heterologous	3	No	Anti-RBD IgG	2 weeks
Cheng CC et al., 2022 [25]	142	72.6 (61.5–80.6)	61.2	HD	BNT162b2 or mRNA-1273	Heterologous	3; 4	Mixed	Neutralizing antibodies	4 weeks
Cheng CY et al., 2022 [26]	19	60.84 ± 13.36	57.9	HD 13; PD 6	mRNA-1273	Heterologous	3	No	Anti-RBD IgG	2–3 weeks
Chinnadurai et al., 2022 [27]	12	61 (49–72)	64.0	HD	BNT162b2	Homologous or heterologous	3	No	Anti-S IgG	4 weeks
Cinkilic O et al., 2022 [28]	19	NR	NR	HD	BNT162b2	Homologous	4	No	Anti-S IgG	4–6 weeks
Davidovic T et al., 2022 [29]	36	66.9 ± 15.9	66.7	HD	Viral Vector Ad26COVS1	Heterologous	3	No	Anti-RBD IgG; IFNγ	4 weeks
Einbinder Y et al., 2022 [30]	106	70.5 ± 14.2	67.0	HD 75; PD 31	BNT162b2	Homologous	3; 4	No	Anti-S IgG	2–3 weeks
Espi M et al., 2022 [31]	75	69.6 ± 15.0	42.1	HD	BNT162b2	Homologous	3	Mixed	Anti-RBD IgG; IFNγ	2 weeks
Gallego-Valcarce E et al., 2022 [32]	178	68.7 ± 14.5	63.5	HD	BNT162b2 or mRNA-1273	Homologous	3	No	Anti-RBD IgG	4 weeks
Garcia P et al., 2022 [33]	2720	NR	NR	HD; PD	BNT162b2 or mRNA-1273	Homologous	3	Mixed	Anti-RBD IgG	2 weeks
Herman-Edelstein et al., 2022 [34]	100	72 ± 12	70.0	HD	BNT162b2	Homologous	3	Mixed	Anti-RBD IgG; IFNγ	2–3 weeks
Housset P et al., 2022 [35]	68	66 (53.8–76.3)	65.0	HD 34; PD 34	BNT162b2	Homologous	3	No	Anti-S IgG	4 weeks
Housset P et al., 2022 [36]	45	72 (56–79)	57.8	HD 17; PD 18	BNT162b2 or mRNA-1273	Homologous	4	No	Anti-S IgG	4 weeks
Kitamura M et al., 2022 [37]	468	69 ± 11	65.0	HD	BNT162b2	Homologous	3	No	Anti-S IgG	3 weeks
Kohmer N et al., 2022 [38]	148	69.6 ± 14.2	56.7	HD 134; PD 14	mRNA-1273	Homologous	3	No	Anti-S IgG	4 weeks
Mosconi G et al., 2022 [39]	109	69.2 ± 13.5	70.1	HD 105 PD 4	BNT162b2 or mRNA-1273	Homologous	3	NR	NR	NR
Patyna S et al., 2022 [40]	37	62 (52–72.5)	69.0	HD	mRNA-1273	Heterologous	3	No	Anti-RBD IgG; IFNγ; IL2	2 weeks
Quiroga B et al., 2022 [41]	451	NR	NR	HD	BNT162b2 or mRNA-1273	Homologous	3	No	Anti-S IgG	4 weeks
Robert T et al., 2022 [42]	18	68.9 ± 13.7	60.0	HD	BNT162b2	Homologous	3	No	Anti-S IgG	4 weeks
Shashar M et al., 2022 [43]	66	75 ± 10.2	59.1	HD	BNT162b2	Homologous	3	No	Anti-S IgG	2–3 weeks
Simon B et al.2022 [44]	60	66 (34–83)	71.7	HD	mRNA-1273	Heterologous	3	No	Anti-RBD IgG; IFNγ;	6 weeks
Tillmann FP et al., 2022 [45]	153	67.4 ± 15.8	60.8	HD	BNT162b2	Homologous	3	No	Anti-S IgG	4 weeks
Verdier JF et al., 2022 [46]	96	71.1 ± 13.1	72.5	HD	BNT162b2	Homologous	3	No	Anti-S IgG	4 weeks

HD, hemodialysis; PD, peritoneal dialysis; KRT, kidney replacement therapy; IgG, immunoglobin G; NR, not reported; RBD, receptor-binding domain; IFNγ: interferonγ; IL2: interleukin2; Homologous vaccinations, the same immunogen vaccination regimen; Heterologous vaccinations, the different immunogen vaccination regimen.

## Data Availability

The datasets generated during and/or analyzed during the current study are available from the corresponding author upon reasonable request.

## Supplementary Materials

The following supporting information can be downloaded at: https://www.mdpi.com/article/10.3390/vaccines10122070/s1, Figure S1: Study selection process. Figure S2: Risk ratios (RRs) for humoral immunogenicity rates associated with dialysis vs. non-dialysis groups. Figure S3: RRs for a SARS-CoV-2 breakthrough infection, all-cause death, and admission in dialysis patients after three doses of the vaccine vs. two doses of the vaccine. Figure S4: Forest plots demonstrating adverse events associated with the SARS-CoV-2 vaccine in patients receiving dialysis.
