# Supplementary figures and images for: Immunogenicity Rates after SARS-CoV-2 Three-Dose Vaccination in Patients under Dialysis: A Systematic Review and Meta-Analysis
Source: Vaccines (Basel). 2022 Dec 2;10(12):2070. doi: 10.3390/vaccines10122070 (PMC9782384; doi:10.3390/vaccines10122070)

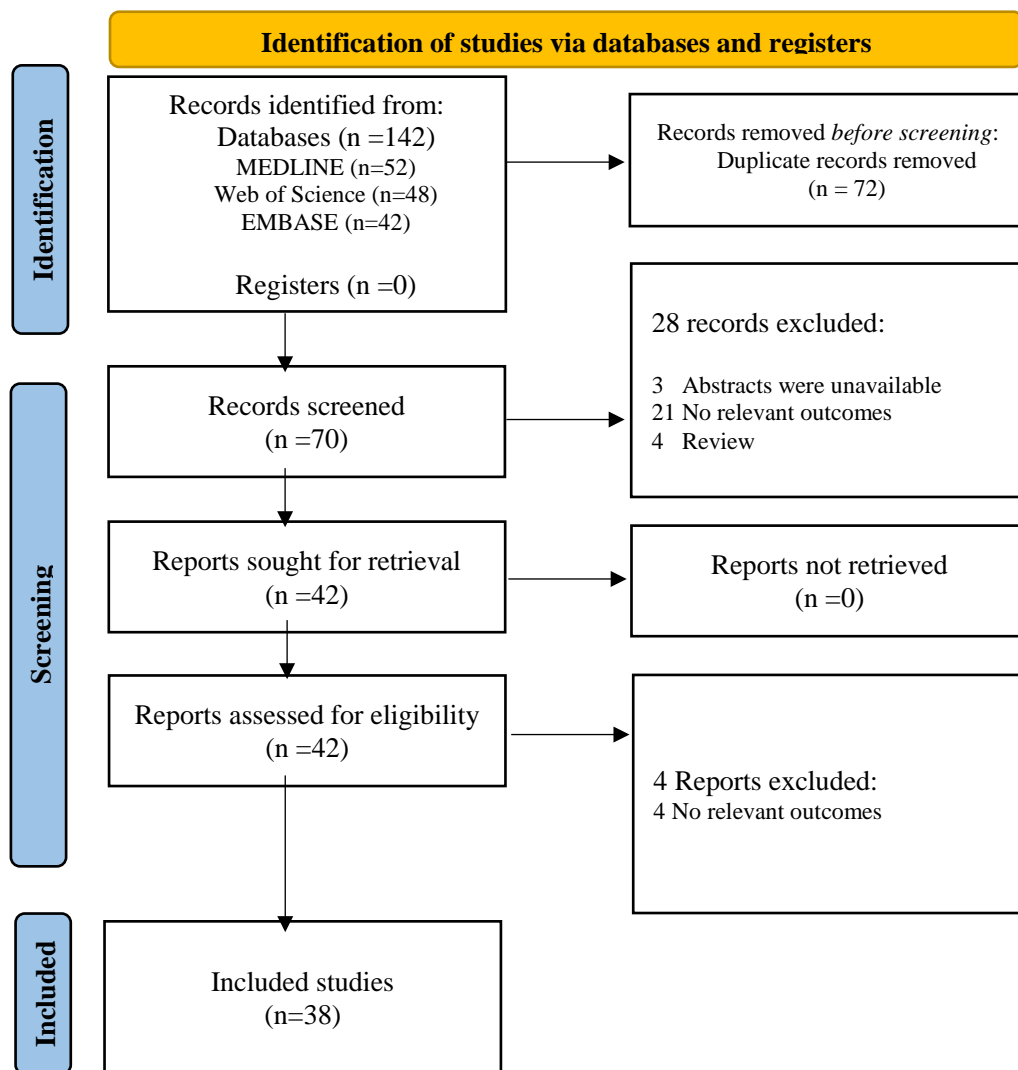

**Figure S1 PRISMA 2020 flow diagram**

Supplement: Supplementary file 1 [file vaccines-10-02070-s001.zip › Figure S1.pdf]

NOTE: Weights are from random effects analysis

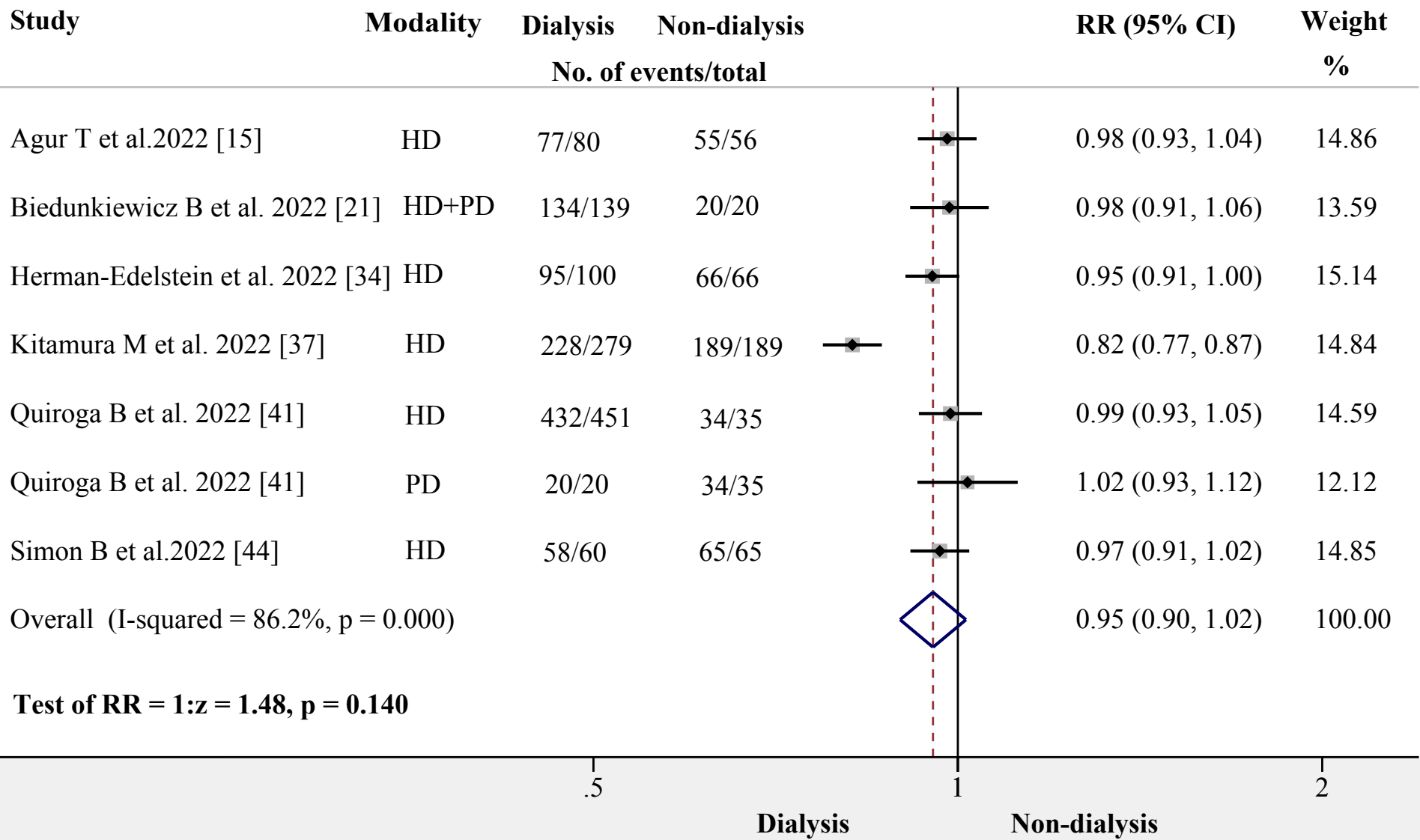

Supplement: Supplementary file 1 [file vaccines-10-02070-s001.zip › Figure S2.pdf]

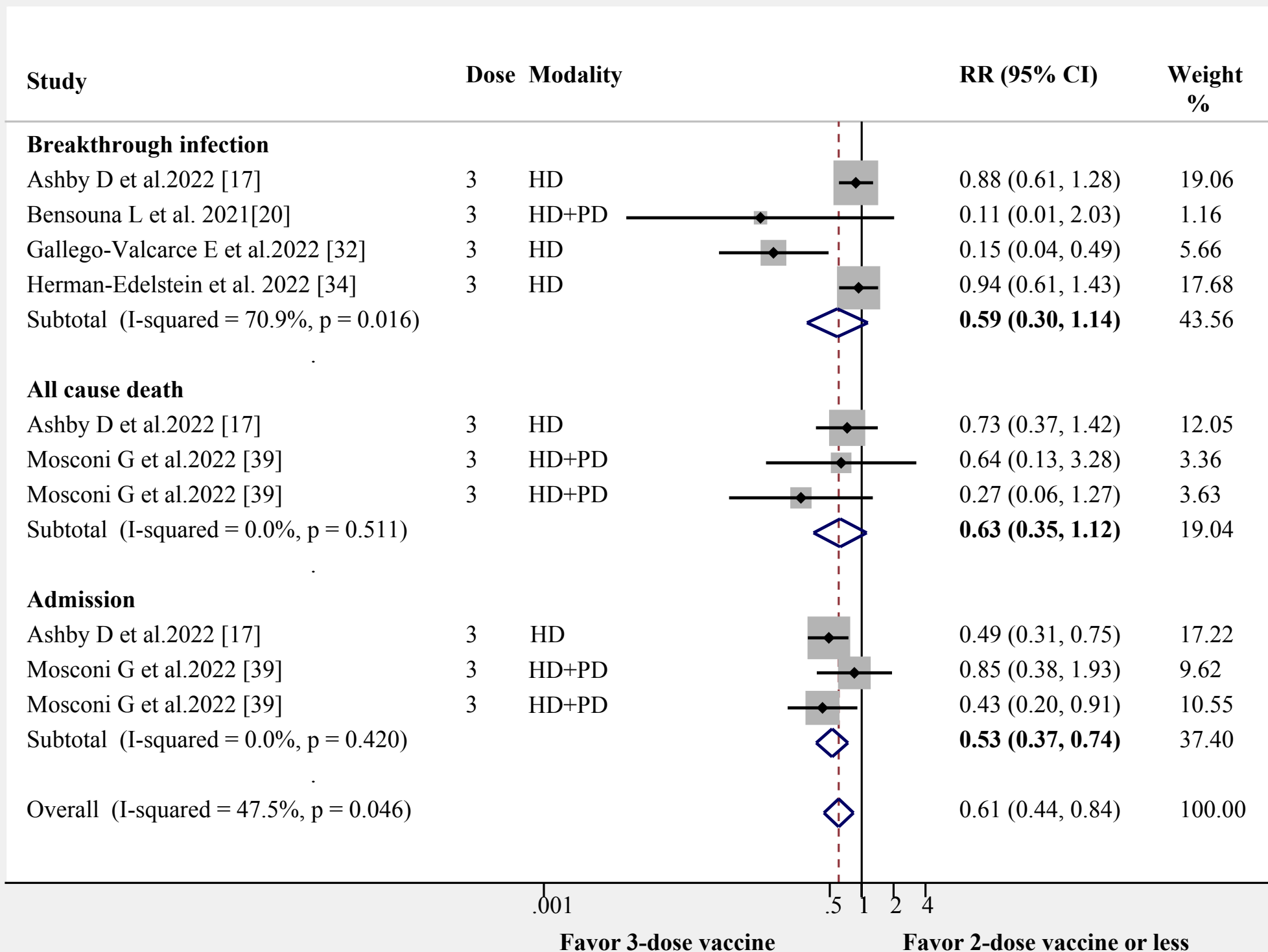

Supplement: Supplementary file 1 [file vaccines-10-02070-s001.zip › Figure S3.pdf]

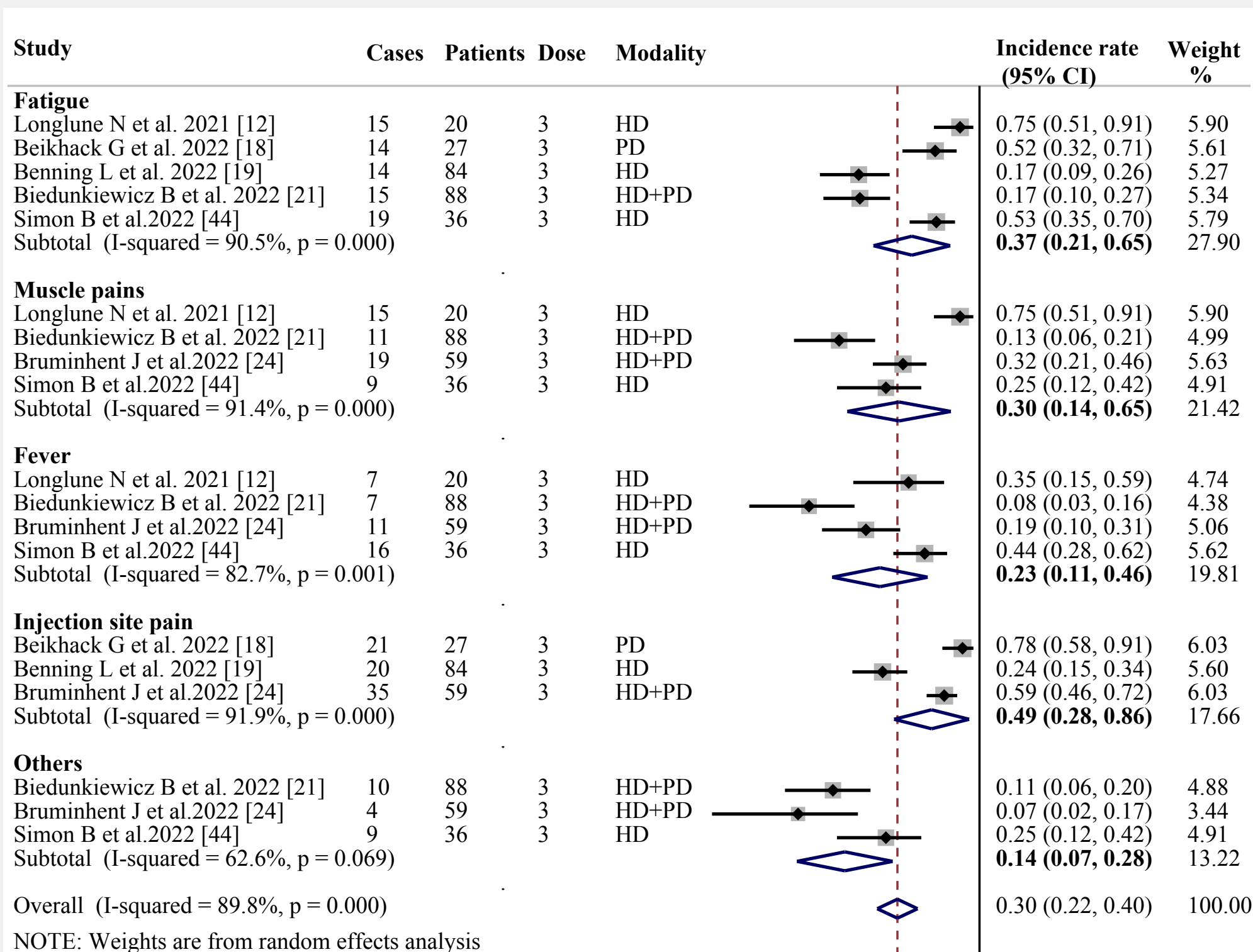

.01 .4 .6 .81

Incidence rate (95%CI)

Supplement: Supplementary file 1 [file vaccines-10-02070-s001.zip › Figure S4.pdf]
